# Global DNA methylation and telomere length as markers of accelerated aging in people living with HIV and non-alcoholic fatty liver disease

**DOI:** 10.1186/s12864-023-09653-2

**Published:** 2023-09-23

**Authors:** Elena Moreno, Javier Martínez-Sanz, Rosa Martín-Mateos, Jorge Díaz-Álvarez, Sergio Serrano-Villar, Diego Burgos-Santamaría, Laura Luna, María Jesús Vivancos, Ana Moreno-Zamora, María Jesús Pérez-Elías, Santiago Moreno, Fernando Dronda, María Luisa Montes, Matilde Sánchez-Conde

**Affiliations:** 1 Department of Infectious Diseases, Hospital Universitario Ramón Y Cajal, Instituto Ramón Y Cajal de Investigación Sanitaria (IRYCIS), 28034 Madrid, Spain; 2 CIBER de Enfermedades Infecciosas (CIBERINFEC), Instituto de Salud Carlos III, 28029 Madrid, Spain; 3 Department of Gastroenterology and Hepatology, Metabolic Liver Disease Clinic, Hospital Universitario Ramón Y Cajal, Instituto Ramón Y Cajal de Investigación Sanitaria (IRYCIS), 28034 Madrid, Spain; 4 Universidad de Alcalá, 28871 Madrid, Spain; 5 Internal Medicine Service, Hospital Universitario La Paz. IdiPAZ, 28046 Madrid, Spain

**Keywords:** DNA methylation, MAFLD, PLWH

## Abstract

Metabolic-dysfunction-associated fatty liver disease (MAFLD) is a comorbidity that generally increases in people living with HIV (PLWH). This condition is usually accompanied by persistent inflammation and premature immune system aging. In this prospective cohort study, we describe a straightforward methodology for quantifying biomarkers of aging, such as DNA methylation and telomere length, in PLWH and in the context of another relevant condition, such as MAFLD. Fifty-seven samples in total, thirty-eight from PLWH and nineteen from non-PLWH participants with or without MAFLD, were obtained and subjected to DNA extraction from peripheral blood mononuclear cells (PBMCs). Global DNA methylation and telomere length quantification were performed using an adapted enzyme-linked immunosorbent assay (ELISA) and qPCR, respectively. The quantification results were analysed and corrected by clinically relevant variables in this context, such as age, sex, and metabolic syndrome. Our results show an increased association of these biomarkers in PLWH regardless of their MAFLD status. Thus, we propose including the quantification of these age-related factors in studies of comorbidities. This will allow a better understanding of the effect of comorbidities of HIV infection and MAFLD and prevent their effects in these populations in the future.

**Supplementary Information:**

The online version contains supplementary material available at 10.1186/s12864-023-09653-2.

## Introduction

People living with HIV (PLWH) have a high incidence of issues resulting from metabolic dysfunction-associated fatty liver disease (MAFLD), which can be even more concerning than in other populations [1]

In addition, PLWH faces adverse effects of antiretroviral therapy (ART) and chronic inflammation caused by persistent infection and chronic treatment [2, 3]. Another component associated with HIV infection is acceleration of the biological clock. Biological clocks have been used to understand the effects of various environmental factors on human health. Acceleration of biological age is higher in PLWH than in non-PLWH [4–7]. This is important because it could bring age-related comorbidities of PLWH forward by up to a decade, thereby decreasing the life expectancy of this population. Aging is an important factor in PLWH, because it increases inflammation and immune activation, and it can be influenced by several components [8–10]. Moreover, age-related illnesses in PLWH, including metabolic diseases, are closely related to the associated heightened levels of systemic inflammation and immune activation, known as “inflamm-aging” [11]. A correlation has been shown between metabolic syndromes and the use of antiretrovirals, particularly protease inhibitors (PI) or nucleoside analogue reverse transcriptase inhibitors (NRTI) [12]. For example, Tenofovir has been directly associated with age acceleration by shortening telomere length [13–15]. Specifically, the markers that have been shown to be most strongly associated with steatosis and premature aging are mediated by altered tissue homeostasis maintenance in PLWH. This causes systemic inflammatory responses, increasing the levels of several systemic markers of inflammation, including TNF-α and interleukin such as IL-6 and C-reactive protein [10]. Immune dysregulation, immune cell senescence, and chronic inflammation are found even in ART-treated PLWH and have been also described as important mechanisms for aging [11, 16].

Fatty liver disease is a broad term that encompasses two main conditions: Non-Alcoholic Fatty Liver Disease (NAFLD) and Alcoholic Fatty Liver Disease (AFLD). Recently, NAFLD has been replaced by MAFLD to highlight the metabolic aspects of the condition [17]. This term aims to encompass a wider range of patients who have fatty liver disease with metabolic risk factors, even if they do not strictly fit the previous criteria used for NAFLD diagnosis. However, metabolic syndrome is not a specific liver condition, but rather a constellation of metabolic abnormalities that can contribute to various health issues, including liver diseases like NAFLD/MAFLD. Metabolic-dysfunction-associated fatty liver disease by itself has become a public health concern, since it is one of the main causes of liver transplants in Western countries [1]. Secondary long-term metabolic complications derived from MAFLD may seriously impact the health and quality of life of patients. Although some non-invasive imaging techniques have been established for the diagnosis of MAFLD, early detection remains challenging [18, 19]. Thus, MAFLD is now considered a health concern, especially in occidental countries, owing to its relationship with metabolic syndrome, diabetes, and high BMI [20]. Furthermore, MAFLD is one of the main comorbidities and mortality factors worldwide in PLWH [21, 22], although studies on MAFLD pathogenesis in PLWH are scarce. There is increasing evidence that the relationship between aging and metabolic dysregulation contributes to fatty liver disease/MAFLD [23–25]. The MAFLD condition may influence the aging process through various mechanisms, such as enhancing chronic inflammation [26, 27], establishing metabolic dysfunctions [20, 28, 29], promoting cellular damage, such as the impairment of mitochondrial functions which leads to increased production of reactive oxygen species (ROS) [30–32] or by accelerating telomere shortening [33, 34].

One method of analysing the effect of MAFLD on health is the quantification of DNA methylation. An inverse correlation between global DNA methylation and disease progression in individuals with MAFLD has been demonstrated in both mouse and human studies [35–37], even describing the role of stress-induced senescence during steatosis development [38]. Other factors, such as mitochondrial DNA damage [32], have been related to MAFLD in animals [24]. In humans, it has been shown that MAFLD patients show epigenetic alterations and age acceleration which can be quantified by analysing cytosine methylation [39, 40]. In addition, studies have investigated how alterations in epigenetics significantly contribute to MAFLD development and have even validated the measurement of DNA methylation as a prognostic marker of aging [41] and liver fibrosis in non-alcoholic fatty liver disease [36, 42, 43].

Lifestyle, environment, and other factors can influence HIV and MAFLD pathogenesis. Epigenetic modifications in response to environmental exposures have been previously described and their relationship with MAFLD has been recently reviewed [44, 45]. Related to HIV infection, differential DNA methylation has been previously described, and epigenetic clocks have been established to understand this difference in PLWH [6, 26, 46, 47]. Furthermore, as determined previously by our group, there are differences in the plasma-free fatty acid profiles and in some genetic variants of PLWH [48]. Therefore, quantifying these senescence markers should be a priority because of the likelihood of unforeseen events arising in this population.

Different methods have been used to measure telomere length; however, quantitative PCR (qPCR) has proven to be a reliable method that allows the use of small amounts of starting material and large amounts of samples in less time and simpler procedures [49–52]. Regarding DNA methylation, different methodologies can be used to quantify it. However, most of these methodologies, although they are more informative and can assist in understanding other phenomena, such as biological clocks, are also more complicated to use on a regular basis, especially in locations with limited time and resources.

As both DNA methylation and telomere length are two relevant markers of biological age acceleration [37, 53] and the side effects of HIV infection and MAFLD persistence, we consider that they should be taken into consideration in studies related with these populations. Thus, this study aimed to show the importance of HIV infection in the biological aging process in PLWH with MAFLD by using straightforward methodologies to quantify biomarkers of aging and reinforce their broad study.

## Results

### Characteristics of the study population

In this study, we analysed samples from 49 participants diagnosed with MAFLD, of whom 30 (61%) were PLWH (MAFLD^+^ & HIV^+^) and 19 (39%) were HIV-uninfected (MAFLD^+^), in addition to 8 control samples from PLWH that were not diagnosed with MAFLD (HIV^+^).

Participant demographic information is provided in Table 1. In summary,PLWH were younger, with a higher percentage of females, lower BMI, and a lower percentage of diabetes mellitus (DM) and metabolic syndrome. The severity of MAFLD between the group of patients with MAFLD and the group of PLWH was significantly different. There was, however, no difference between PLWH regardless of whether they had MAFLD or not (Table 1). Regarding diet, except for lower dairy consumption and higher alcohol consumption, there were comparable results in PLWH. At inclusion, all PLWH participants were receiving antiretroviral therapy (94% under an integrase-strand-inhibitor-based regimen) for an average of 6 years while maintaining a suppressed viral load (see Table 1).

**Table 1:** Clinical characteristics of the studied populations

	**MAFLD (*n* = 19)**	**PLWH (*n* = 8)**	**MAFLD & PLWH (*n* = 30)**	***p*-value**^**1**^
**Age,**	60.15 (56.57—70.71)			0.220
**median (IQR)**		55.55 (50.40—57.17)	55.78 (48.96—60.44)	
**Sex, n (%)**				**0.027**
Male	9 (47.37)	1 (12.5)	4 (12.33)	
Female	10 (52.63)	7 (87.50)	26 (86.67)	
**Race, n (%)**				0.264
American	1 (5.26)	1 (14.29)	9 (30)	
Asian	0 (0.00)	0 (0.00)	1 (3.33)	
Caucasian	14 (73.68)	4 (57.14)	13 (43.33)	
ND^b^	4 (21.05)	2 (28.57)	7 (23.33)	
**Country, n (%)**			14 (50)	0.686
Spain	13 (68.42)	5 (71.43)	12 (42.84)	
America	3 (31.57)	1 (14.29)		
(Central and South)			2 (7.14)	
ND^b^	4 (21.05)	1 (14.29)		
Body Mass Index (BMI)	32.8 (31–35.5)	22.6 (21.1–26.3)	27.3 (24.9–28)	**0.0001**
Transient Elastography kPa	7.2 (6.1–9.5)	4.2 (3.9–4.4)	4.1 (3.9–4.3)	**0.0001**
Transient Elastography CAP	312 (273–359)	210 (134–230)	275 (234–288)	**0.015**
**HCV**				0.818
No	1 (100)	7 (87.50)	25 (83.33)	
RVS	0 (0.00)	1 (12.50)	2 (6.67)	
Cleared	0 (0.00)	0 (0.00)	3 (10.00)	
**HBV**				0.371
No	1 (100)	2 (25.00)	7 (23.33)	
Cleared	0 (0.00)	2 (25.00)	14 (46.67)	
Vaccinated	0 (0.00)	4 (50.00)	9 (30.00)	
**Alcohol consumption**^a^				0.717
No	17 (89.47)	6 (75.00)	22 (73.33)	
Yes	1 (5.26)	1 (12.50)	4 (13.33)	
ND^b^	1 (5.26)	1 (12.50)	4 (13.33)	
**Tobacco use**				0.393
Currently	2 (10.53)	2 (25.00)	5 (16.67)	
Past	3 (15.79)	2 (25.00)	12 (40.00)	
Never	12 (63.16)	4 (50.00)	10 (33.33)	
ND^a^	2 (10.53)	0 (0.00)	3 (10.00)	
**Statins**				1.00
Yes	9 (47.37)	4 (50.00)	13 (43.33)	
**Metformin**				**0.001**
Yes	9 (47.37)	0 (0.00)	2 (6.90)	
**Insulin**				0.474
Yes	1 (5.26)	0 (0.00)	0 (0.00)	
**GLP1**^c^				0.474
Yes	1 (5.26)	0 (0.00)	0 (0.00)	
**DPP4**^d^				0.082
Yes	3 (15.79)	0 (0.00)	0 (0.00)	
**Fibrates**				0.805
Yes	2 (10.53)	0 (0.00)	2 (6.67)	
**Ezetimibe**				0.684
Yes	2 (11.11)	1 (12.50)	2 (6.67)	
**Hypertension**				**0.009**
Yes	11 (57.89)	0 (0.00)	9 (30.00)	
**DM**^e^				**0.000**
Yes	11 (57.89)	0 (0.00)	3 (10.00)	
**Metabolic Syndrome**				
Yes	15 (78.95)	0 (0.00)	6 (20.00)	**0.000**

^1^Fisher’s exact test

^a^Yes = lower than 30gr/day for men and 20gr/day for women

^b^ND = Not defined

^c^GLP1 = hormone glucagon-like peptide-1

^d^DPP4 = dipeptidyl peptidase 4 inhibitors

^e^DM = diabetes mellitus

### Cytosine global methylation as a marker of senescence in PLWH with MAFLD

**Fig. 1:**
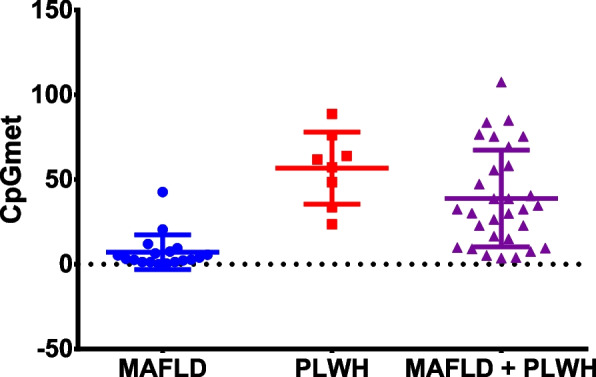
Quantification of cytosine methylation using ELISA assay. Levels of methylation at CpG sites (CpGmet) were measured and calculated as detailed in the Methods section. The calculated results from the duplicates assayed are shown here at each point, and the samples were divided into three groups according to the patient: 19 patients with MAFLD (blue), 8 PLWH (red), and 30 PLWH and MAFLD (purple). The limit of detection was established above the last point of the standard curve (0). Significant differences obtained by linear regression analysis after adjusting by age, sex, and metabolic syndrome (Least Square Mean difference) comparing group to group were significant for the following comparisons: MAFLD vs PLWH (*p* = 0.005, B0 = 37.81) and MAFLD vs MAFLD + PLWH (*p* = 0.03, B0 = 22.06) (Figure S1)

Cytosine methylation is one of the main markers used to quantify DNA methylation which is the main epigenetic parameter related to age, substances, and diseases. In this study, we quantified the total amount of 5-methylcytosine in DNA samples (levels of CpG methylation per sample) using a straightforward approach based on a colorimetric assay. Analysis of the results from 57 samples revealed significant differences, showing the lowest methylation levels in non-PLWH with MAFLD (Fig. 1).

### Telomere length as a marker of senescence in PLWH with MAFLD

**Fig. 2:**
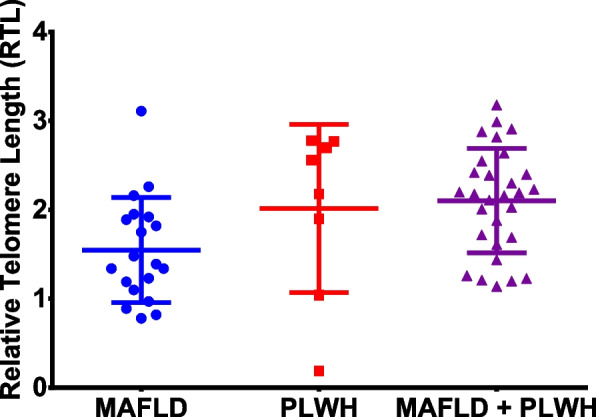
Relative telomere length quantification by qPCR. Telomere length was measured by qPCR and calculated as described in the Methods section. Calculated relative telomere lengths are depicted by dividing the samples into three groups according to the patients: 19 patients with MAFLD (blue), 8 PLWH (red), and 29 PLWH and MAFLD (purple). Differences obtained by linear regression analysis adjusted by age, sex, and metabolic syndrome (Least Square Mean difference) were not significant for any of the group to group comparisons analysed for this quantification (Figure S1)

Telomere length has been widely used as a marker of age acceleration. However, different methodologies can be used for this quantification. In this study, we used a previously described qPCR protocol based on the detection of specific sequences in the telomere region. The results showed the greatest differences when comparing the PLWH and MAFLD groups (Fig. 2), although the differences were not statistically significant.

### Further statistical analyses

**Fig. 3:**
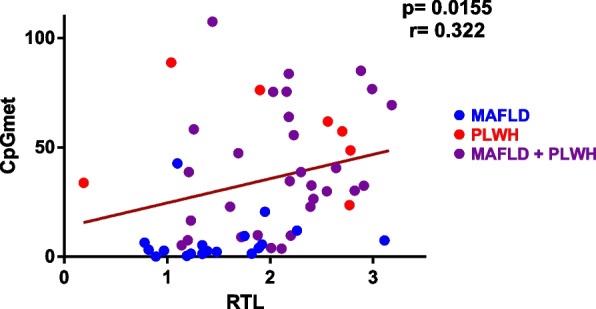
Correlation analysis between methylation data and telomere length data. Linear regression and Pearson correlation was calculated for the 57 samples together since the separated groups had a reduced sample size that could alter the meaning of the statistics. However, the three different groups of the samples have been depicted in different colours (MAFLD, blue; PLWH, red and MAFLD + PLWH, purple) for clarification. The purpose of this analysis is to understand the relationship between the quantification of these two markers in general, but specifically in the context of the methods used in this study

Different results have been reported in studies on the relationship between methylation and telomere length. In fact, a method to evaluate epigenetic modifications related with telomere length was described [41]. Thus, in our study we aimed to explore whether these two biomarkers of aging were correlated. We fitted the data from both analyses and adjusted them by linear regression. Pearson correlation analysis for methylation data (CpGmet) and telomere length (RTL) showed a moderate correlation (*p* = 0.0155, *r* = 0.32) (Fig. 3).

## Discussion

In this prospective study, we analysed the effect of HIV and MAFLD in biomarkers of aging, such as DNA methylation and telomere length, by using straightforward methods. Aging has been shown to be clinically relevant for PLWH because it is related to inflammation and immune activation; therefore, the term ‘inflamm-aging’ is used to describe this effect [10, 11]. Telomere length has been widely used as a marker of aging, although its clinical use as the only relevant marker of aging has been questioned. Thus, other factors, such as frailty and epigenetic markers, have been proposed as complementary markers [54, 55]. Epigenetic regulation, such as DNA methylation, has also been linked to the inflammatory status of PLWH [46, 47, 56]. Furthermore, the role of epigenetics in the pathogenesis of MAFLD has been described to explain the effects of lifestyle and environmental factors [45].

Increased methylation in PLWH has been observed within three years after initial HIV infection, although there is some decrease associated with the use of antiretroviral therapy [15, 57]. Furthermore, alterations in DNA methylation have been associated with MAFLD susceptibility [58], although the fact that it may be down- or upregulated depends on the gene being studied [56]. These differences have been addressed by other studies that quantified methylation in CpGs at specific loci [59]. However, these technologies are complex and expensive; therefore, they may not easily be implemented in clinical practice. In this study, we addressed this question by quantifying the level of global DNA methylation using an affordable and straightforward approach. The results showed that CpG methylation levels remained high in the presence of HIV infection, independently of their MAFLD status. Although different associations have been described, previous studies have also pointed to an association between DNA methylation and HIV susceptibility [46, 60, 61]. However, the quantification of global DNA methylation by ELISA, has yielded inconsistent results in PLWH. Some studies have shown an increase [61–63] while others have reported a decrease [46, 64] in DNA methylation in PLWH, although it is important to note that these studies used different kits for quantification, making it difficult to compare the results. It has been shown that DNA methylation differs between different immune cell types [65]. In the particular case of HIV, DNA methylation has been related to the CD4/CD8 ratio, viral load and response to treatment [61, 66]. However, more information is needed to take conclusions about this topic and further studies should be performed to understand the effect of aging in PLWH.

Regarding telomeres, only some changes associated with the enzyme Telomerase reverse transcriptase, which is involved in the maintenance of telomeres, have been directly linked to MAFLD-associated conditions [67, 68]. However, in PLWH, shorter telomeres have been previously described, although this has been shown to be controlled by antiretroviral therapy, probably due to the decrease in immune activation [12–14, 69]. Here, we decided to study the telomere length relationship with PLWH and MAFLD using relative quantification (qPCR) because it is a feasible technique and can be useful in different contexts. However, following this approach, in our setting, only a trend of increased telomere length without statistical significance was found in PLWH regarding MAFLD status. This could be due to the fact that, although qPCR is one of the main methods used to quantify telomere length because it allows small amounts of DNA to be used, is less labour-intensive, and can be used in high-throughput settings, it also has its limitations. Lacking reference standards, variation between batches, and recognition of mean length measures instead of individual telomeres or ends must be considered in order to interpret the lack of significance when using this technique [50, 52].

Importantly, in this study, some confounding variables related to HIV infection and MAFLD development that could be important for the analysis of DNA methylation and telomere length, were also analysed. In the two PLWH groups, there was a significantly higher number of women, with a lower BMI, hepatic stiffness (KPa), liver steatosis measured by CAP, diabetes mellitus, and prevalence of metabolic syndrome. Furthermore, the two groups of PLWH participants were five years younger on average, although this difference was not significant. Therefore, in addition to adjusting the model for sex and age, we included metabolic syndrome as an adjustment variable, considering it as a proxy for the others, to avoid overfitting and collinearity. In addition, multivariate models were fitted, with similar estimates, adjusting for the presence of DM or arterial hypertension. After adjustment, we can assume that the groups were comparable, as the results were similar to the unadjusted model, except for telomeric length, which did not reach statistical significance, probably due to the small sample size.

DNA methylation and telomere length as biomarkers of aging have shown different levels of correlation when studied previously, especially when adjusted for age [54, 70]. In our study, these factors showed a moderate correlation which could be due to differences in methodologies applied in comparison with previous studies. It has been previously shown that chronic inflammation and immune activation are typically present in PLWH, even if they are under ART, and provoke premature aging, significantly affecting their quality of life [10, 11]. Consequently, our analysis of PLWH showed an increasing trend in DNA methylation and telomere length. A correlation between these two factors using these two specific methods has not been shown previously, and it may be relevant to demonstrate that these techniques can be accessible for daily use.

The limitations of our study include the lack of a control group of healthy patients. However, since this study focused on the methodology applied to understand the role of MAFLD in PLWH, we used samples from patients with MAFLD as a reference group as some other groups have previously done to study hepatic steatosis [71]. Regarding the quantification techniques, limitations are inherent for each method. For telomere length, there is no consensus about the best protocol to perform these quantifications; therefore, we followed the literature and simplified the method as much as possible because we only aimed to determine relative differences. Regarding methylation, there are of course more sophisticated methods of quantification, but these are more complicated and expensive, and this study aimed to find an affordable approach to study these factors and determine their relevance in the clinical setting.

## Conclusion

Although this was an exploratory study to test straightforward techniques to quantify DNA methylation and telomere length in a limited number of samples from a very specific population, it provides information about the utility of these facile approaches for testing clinical variables. Furthermore, our findings underline the importance of HIV infection as an accelerating factor for biological aging even if complete virological control has been achieved and independently of other factors or comorbidities, such as MAFLD status.

## Materials and methods

### Study design and sample collection

This was a prospective cohort study at Hospital Universitario Ramón y Cajal in Madrid, Spain from January 2018 to December 2018. non-PLWH participants diagnosed with MAFLD were recruited at the Metabolic Liver Disease Clinic. PLWH participants receiving suppressive antiretroviral therapy were recruited at the HIV Clinic. We included those who presented with elevated liver enzymes for at least two determinations, separated by six months. Any transaminase (GGT, ALT, AST) level above the upper limit of normal in our laboratory was considered. PLWH were receiving antiretroviral therapy for at least 1 year with undetectable viremia in the last 6 months. All participants underwent abdominal ultrasound and a screening analysis for liver disease. Based on our previously published data, diagnosis of MAFLD was established by Transient Elastography (CAP), ultrasound confirmation of steatosis, and exclusion of other aetiologies of chronic liver disease. Metabolic syndrome was defined according to the National Cholesterol Education Program (NCEP)’s third report [72] and the criteria defined by Eslam et al. [73] based on evidence of hepatic steatosis, in addition to one of the following three criteria, namely overweight/obesity, presence of type 2 diabetes mellitus, or evidence of metabolic dysregulation. In addition, diagnosis of MAFLD was screened by non-invasive serological markers (triglyceride and glucose index [TyG], fatty liver index [FLI]) in those participants who met the inclusion criteria [74]. Exclusion criteria included active viral hepatitis; alcohol abuse (defined by > 30 g daily in men and > 20 g daily in women; lower consumption was allowed); cocaine, heroin, or designer drug abuse; other known liver diseases (autoimmune, genetic, drug-related); isolated alkaline phosphatase alteration; recent drug toxicity; the impossibility of cannulating a peripheral bloodline if a liver biopsy was required; pregnancy or desired pregnancy; decompensated liver disease or hepatocarcinoma; and any other comorbidity that, at the investigator’s discretion, could prevent correct compliance with the study protocol. Clinical information was collected through the clinical interview at the baseline visit of the study, together with a review of the patient’s medical history recorded in the electronic medical record. The study was approved by the Institutional Review Boards of the Carlos III Health Institute, Madrid, Spain (Project PI 17/01717), and by the Ethics Committee at the University Hospital Ramón y Cajal (ceic.hrc@salud.madrid.org, Approval Number 097/17). All patients gave written informed consent before the initiation of the study procedures. All methods were performed following the relevant guidelines and regulations. Peripheral blood mononuclear cells (PBMCs) from patient blood samples collected in EDTA tubes were isolated using Ficoll-Hypaque density gradient (Comercial Rafer S.L., Zaragoza, Spain) and following multiple previously described standard methods.

### Methylation analysis

For the determination of the global DNA methylation in the isolated PBMCs from patients, total DNA was extracted by using the commercially available QIAAMP DNA MINI KIT (Qiagen, Hilden, Germany) following the manufacturer´s instructions. Then, evaluation of the global DNA methylation of liver-derived DNA was performed by measuring the concentration of 5-methylcytosine (5-mC) using a commercially available kit (5-mC DNA ELISA Kit, Zymo Research, Irvine, CA, USA) following the manufacturer’s instructions. Each sample was assayed in duplicate, seeking an equal representation of the study groups. After reading absorbance at 405 nm (OD), a standard curve was generated using a logarithmic curve, and %5-mC values were extrapolated from the curve (in %5-mC). Data obtained from the 0% value of the standard curve were used as negative control and subtracted from all the positive values. Then, to calculate the level of CpG methylation, the observed ODs extrapolated from the logarithmic standard curve were multiplied by the fold difference CpG density between control DNA derived from E. coli (1.00) and the standard human genome hg19 (8.167). Data were provided by the manufacturer of the kit. Final data were represented in a dot-plot comparing the three groups.

### Telomere length analysis

For the determination of the average telomere length in total DNA extracted from PBMCs of patients (as indicated previously), we performed a qPCR-based method previously described [21]. After DNA extraction, the purity of DNA was evaluated by detection of the absorbance 260 nm (A260)/absorbance 280 nm (A280) ratio in a NanoVue™ (GE), and concentration was quantified in a Qubit® Fluorometer with a Qubit® dsDNA BR Assay Kit. Then, the qPCR mix was prepared with the following amounts per well: 5ul of SYBR® Green Master Mix from Roche (#0470751600), 10 ng of DNA, and primers at 1uM final concentration. DNA samples were assessed in triplicate in 96-well plates for telomere (T) and housekeeping gene 36B4 (S) primers. The primer sequences (5′ → 3′) were:

- tel1b CGGTTTGTTTGGGTTTGGGTTTGGGTTTGGGTTTGGGTT;
- tel2b GGCTTGCCTTACCCTTACCCTTACCCTTACCCTTACCCT;
- 36B4u CAGCAAGTGGGAAGGTGTAATCC;
- 36B4d CCCATTCTATCATCAACGGGTACAA;
- 18S For GTAACCCGTTGAACCCCATT;
- 18S Rev CCATCCAATCGGTAGTAGCG;

Standard DNA, a positive control (commercially available DNA-Human Genomic DNA, Roche), and a non-template control (NTC) were assessed in parallel.

Relative qPCR was carried out on a LightCycler 480 Roche System. The thermal cycling began with the initial polymerase activation step (10 min at 95 °C) and was followed by 40 cycles of 95 °C for 15 s and 60 °C for 1 min. A melting curve analysis was performed to verify the specificity and identity of the products.

For calculations, ∆Ct was calculated for each sample as (Average Ct of 18S)-(Average Ct of telomere) and (Average Ct of 18S)-(Average Ct of 36B4). Since telomeres are present in multiple copies in a cell, as against single-copy genes, ∆Ct value is positive. Then, the relative telomere length (RTL) as ∆∆Ct was calculated by subtracting the ∆Ct for Control DNA (Control DNA Average 36B4 Ct-Control DNA Average Telomere Ct). A higher ∆∆Ct value indicates a longer telomere length. Telomere length has been calculated previously through the measure of the expression of a specific region in the telomere sequence (T) compared to a regular region of a single copy gene (S), based on methods described in previous studies [49–51] to obtain the Relative Telomere Length (RTL).

### Statistical analysis

Baseline characteristics of patients with MAFLD, HIV infection, or both were compared with the Wilcoxon rank-sum test for continuous variables and Fisher’s exact test for categorical variables. All contrasts were two-tailed and a *p* < 0.05 was considered statistically significant. Differences between the groups were compared with linear regression and adjusted by variables relevant for age acceleration (sex, age, metabolic syndrome) (details in Figure S1A and B). A sensitivity analysis was also conducted to eliminate samples from participants with cured HCV, and obtaining similar estimates (details in Figure S1C). Pearson correlation analysis was performed to evaluate the correlation between the two studied variables (methylation and telomere length). Analyses were performed with Stata v. 17.0 (StataCorp LP, College Station, TX, USA). Figures were created using GraphPad Prism v9.2 (GraphPad, La Jolla, CA, USA).

### Supplementary Information

**Additional file 1. **Linear regression analysis tables obtained from STATA.

## Data Availability

Data are available on request. The data presented in this study are available on request from the corresponding author.
