# Supplementary material for: Global DNA methylation and telomere length as markers of accelerated aging in people living with HIV and non-alcoholic fatty liver disease
Source: BMC Genomics. 2023 Sep 23;24:567. doi: 10.1186/s12864-023-09653-2 (PMC10517540; doi:10.1186/s12864-023-09653-2)
Supplement: Supplementary file 1 — Additional file 1. Linear regression analysis tables obtained from STATA. [file 12864_2023_9653_MOESM1_ESM.pdf]

Figure S1

A. Lineal regression analysis result for methylation data adjusted by sex, age and metabolic syndrome:

```
regress met i.group sex age sdmet
```

Source	SS	df	MS
Model	17854.672	5	3570.9344
Residual	27697.0034	50	553.940069
Total	45551.6754	55	828.212281

Number of obs	= 56
F(5, 50)	= 6.45
Prob > F	= 0.0001
R-squared	= 0.3920
Adj R-squared	= 0.3312
Root MSE	= 23.536

met	Coef.	Std. Err.	t	P>|t|	[95% Conf.	Interval]
group						
hiv	37.81065	12.92824	2.92	0.005	11.8435	63.77779
hiv and nash	22.05978	9.776461	2.26	0.028	2.42318	41.69638
sex	6.527869	7.980103	0.82	0.417	-9.50064	22.55638
age	-.0277798	.3430109	-0.08	0.936	-.7167375	.6611779
sdmet	-10.64851	8.40743	-1.27	0.211	-27.53533	6.238307
_cons	14.71444	22.86531	0.64	0.523	-31.21188	60.64076

B. Lineal regression analysis result for telomere lenght data adjusted by sex, age and metabolic syndrome:

```
regress telom i.group sex age sdmet
```

Source	SS	df	MS
Model	4.04674219	5	.809348438
Residual	20.5145704	49	.418664703
Total	24.5613126	54	.454839123

Number of obs	= 55
F(5, 49)	= 1.93
Prob > F	= 0.1057
R-squared	= 0.1648
Adj R-squared	= 0.0795
Root MSE	= .64704

telom	Coef.	Std. Err.	t	P>|t|	[95% Conf.	Interval]
group						
hiv	.4053819	.3554293	1.14	0.260	-.3088801	1.119644
hiv and nash	.5047478	.269778	1.87	0.067	-.0373913	1.046887
sex	-.2792006	.2195564	-1.27	0.209	-.7204157	.1620145
age	-.0065559	.0095627	-0.69	0.496	-.0257728	.0126611
sdmet	-.0796635	.2318935	-0.34	0.733	-.545671	.3863439
_cons	2.199475	.6348337	3.46	0.001	.923729	3.475221

C. Sensitivity analysis eliminating samples from participants with cured HCV, for both, methylation (top) and telomere length (bottom) data: :

met	Coef.	Std. Err.	t	P>|t|	[95% Conf.	Interval]
group						
hiv	48.1607	10.20094	4.72	0.000	27.65034	68.67107
hiv and nash	33.02709	7.021968	4.70	0.000	18.90848	47.1457
_cons	7.883765	5.293007	1.49	0.143	-2.75854	18.52607

telom	Coef.	Std. Err.	t	P>|t|	[95% Conf.	Interval]
group						
hiv	.39	.2862271	1.36	0.180	-.1858146	.9658146
hiv and nash	.4772222	.1987933	2.40	0.020	.0773017	.8771427
_cons	1.5467	.1485159	10.41	0.000	1.247924	1.845476

Figure S1: Linear regression analysis tables obtained from STATA. hiv = PLWH, nash = MAFLD, hiv and nash = PLWH and MAFLD. All the analysis are performed compared to the reference group, MAFLD.
